# Effects of Drugs in Lactation

**Published:** 1881-11

**Authors:** 


### Effects of Drugs in Lactation.

Dr. Dolan, continuing (*Practitioner*, September, 1881) his series of articles on this subject, from which we have already quoted, gives a case where fifteen grains of chloral were given to a patient every few hours before confinement, until seventy-five grains had been taken. No trace of chloral was found in the milk on third day; but Dr. Dolan thinks chloral does have an effect on the milk.

Calabar bean has been suggested as a means of restoring the suppressed secretion of milk. Dr. Dolan tried this remedy, but without effect on the flow of the milk.

Cod-liver oil was given to two nursing women in the dose of one tablespoonful three times a day in a cup of warm milk. The children nursed as usual, and no physiological effect was produced on them. Linseed tea, beef fat, and mutton fat were administered to women with the view of ascertaining what, if any, effect would be produced upon the amount of breast-milk secreted. In two cases no effect was produced.

Castor oil was given to mothers in a number of cases. It always produced a purgative action on the child.

Of cummin, as of other aromatics, Dr. Dolan says, "I believe that I may formulate the law that they all impart a flavor and odor to human milk, without increasing the quantity or improving the quality of the secretion."

Of conium he remarks, "Most of the Umbelliferæ are readily absorbed by the lacteal vessels, and may be easily found in the milk. Conium, from its sedative action and its influence on the nerves of motion, could not be expected to increase the milk-supply. There are reasons, however, for its administration to mothers who are nursing, so that it is important to note how soon, if at all, it appears in the milk, and what dose produces an effect. Conium, praised by Störck for the cure of uterine scirrhus, and by Dr. Tunstall for chronic inflammation of the womb, is an excellent sedative for back-ache and for the sexual organs. It must be given until its physiological effects are produced, and this means a dose of the succus conii B. P. of two or three drachms."

He administered two-drachm doses of the succus conii every three hours to a patient until she had taken twelve drachms. Analysis of the milk then showed the absence of conium.

Digitalis infusion, in half-ounce doses, every six hours, was given in three cases without any trace of the medicine being found in the milk.

Regarding ergot, Dr. Dolan says his results are negative or uncertain. Two grains every two hours were given to a nursing mother until twelve grains had been administered. The mother thought the child was affected, as it was cross and irritable. No ergot could be found in the milk, although it must be admitted that our present methods of analysis are unsatisfactory.

Of iodide of patassium Dr. Dolan says that in his experience it does not decrease the quantity of milk, although its prolonged use may deteriorate the milk by impoverishing the blood. He found iodine in the milk of a patient who had taken fifteen grains of iodide of potassium every three hours until sixty grains had been taken. A child eighteen months old, who had been given the milk from a woman taking iodide of potassium, showed iodine in the urine.

Of mercury Dr. Dolan's experience is inclusive. In the two cases examined, no trace of mercury was found in the milk.

Opium, when given to the nursing mother in large doses, can be found in the milk. After small doses it cannot be detected.

Phosphorus was given to two nursing women in doses of one-thirtieth of a grain for fourteen days without any trace of the drug being found in milk or urine. The potash salts and quinine do not, in Dr. Dolan's experience, pass into the milk. Rhubarb and senna, however, do, and produce their physiological effect on the nursing child. Sulphur and turpentine, administered internally, affected the milk, the former acting as a mild purgative on the nursing infant. Valerian, dill and copaiba are also excreted by the milk.

